# SCN1A: bioinformatically informed revised boundaries for promoter and enhancer regions

**DOI:** 10.1093/hmg/ddad015

**Published:** 2023-01-28

**Authors:** Susanna Pagni, Helena Martins Custodio, Adam Frankish, Jonathan M Mudge, James D Mills, Sanjay M Sisodiya

**Affiliations:** Department of Clinical and Experimental Epilepsy, UCL Queen Square Institute of Neurology, London WC1N 3BG, UK; Chalfont Centre for Epilepsy, Bucks SL9 0RJ, UK; Department of Clinical and Experimental Epilepsy, UCL Queen Square Institute of Neurology, London WC1N 3BG, UK; Chalfont Centre for Epilepsy, Bucks SL9 0RJ, UK; European Molecular Biology Laboratory, European Bioinformatics Institute, Cambridge, UK; European Molecular Biology Laboratory, European Bioinformatics Institute, Cambridge, UK; Department of Clinical and Experimental Epilepsy, UCL Queen Square Institute of Neurology, London WC1N 3BG, UK; Chalfont Centre for Epilepsy, Bucks SL9 0RJ, UK; Amsterdam UMC, Department of (Neuro) Pathology, Amsterdam Neuroscience, University of Amsterdam, Amsterdam, 1105 AZ The Netherlands; Department of Clinical and Experimental Epilepsy, UCL Queen Square Institute of Neurology, London WC1N 3BG, UK; Chalfont Centre for Epilepsy, Bucks SL9 0RJ, UK

## Abstract

Pathogenic variations in the sodium voltage-gated channel alpha subunit 1 (*SCN1A*) gene are responsible for multiple epilepsy phenotypes, including Dravet syndrome, febrile seizures (FS) and genetic epilepsy with FS plus. Phenotypic heterogeneity is a hallmark of *SCN1A*-related epilepsies, the causes of which are yet to be clarified. Genetic variation in the non-coding regulatory regions of *SCN1A* could be one potential causal factor. However, a comprehensive understanding of the *SCN1A* regulatory landscape is currently lacking. Here, we summarized the current state of knowledge of *SCN1A* regulation, providing details on its promoter and enhancer regions. We then integrated currently available data on *SCN1A* promoters by extracting information related to the *SCN1A* locus from genome-wide repositories and clearly defined the promoter and enhancer regions of *SCN1A*. Further, we explored the cellular specificity of differential *SCN1A* promoter usage. We also reviewed and integrated the available human brain-derived enhancer databases and mouse-derived data to provide a comprehensive computationally developed summary of *SCN1A* brain-active enhancers. By querying genome-wide data repositories, extracting *SCN1A*-specific data and integrating the different types of independent evidence, we created a comprehensive catalogue that better defines the regulatory landscape of *SCN1A*, which could be used to explore the role of *SCN1A* regulatory regions in disease.

## Graphical Abstract

**Graphical Abstract:**
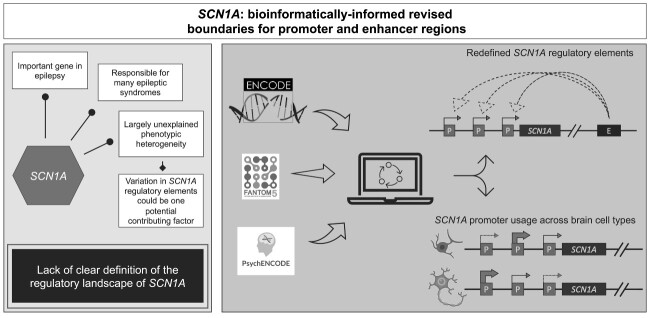

## Introduction

Pathogenic variants in the voltage-gated sodium channel alpha subunit 1 gene (*SCN1A*) are responsible for multiple epilepsy phenotypes, the most well-recognized of which is Dravet syndrome (DS) (1). DS is a developmental and epileptic encephalopathy characterized by early onset drug-resistant epilepsy and neurodevelopmental delay, with subsequent motor and cognitive dysfunction, and variable degrees of intellectual disability (1,2). In addition to DS, the broad phenotypic spectrum of *SCN1A*-related epilepsies includes febrile seizures (FS) alone, genetic epilepsy with FS plus (GEFS+) and other epilepsy syndromes, frequently associated with significant comorbidities (1). Phenotypic heterogeneity is a hallmark of *SCN1A*-related epilepsies, not only because of the variety of epileptic syndromes that can result from a pathogenic variant in *SCN1A* but also because the same *SCN1A* variant can lead to different epileptic disorders and degrees of phenotypic severity (3,4). The causes of this phenotypic variability are yet to be clarified, and multiple factors have been suggested, including the type and location of the *SCN1A* variant, mosaicism of the pathogenic *SCN1A* variant, the presence of variants in other genes and the contribution of the non-coding regulatory genome (4,5).

Gene regulatory elements, including gene promoters and enhancers, play a crucial role in the modulation of gene expression, with established consequences in human disease (6–8). For example, in Parkinson’s disease, variants have been described in an enhancer region of the synuclein alpha (*SNCA)* gene, a key gene in Parkinson’s disease pathogenesis, and shown to modulate gene expression, leading to an increased or decreased risk of developing Parkinson’s disease (7,8). In cancer biology, variation in the telomerase reverse transcriptase (*TERT*) gene promoter has been demonstrated to participate in the tumorigenic process via the creation of novel transcription factor binding sites (TFBSs) that support permanent telomerase expression and capacity for indefinite cell proliferation (9). The status of *TERT* promoter variation as a predictor of prognosis and as a potential therapeutic target is being explored (6).

In the case of *SCN1A*, we have an extensive understanding of its biophysics and how its disruption causes disease, but we know much less about its regulation. There are three known *SCN1A* promoters, which are associated with three untranslated exons (UEs) (h1a, h1b and h1c), each containing a transcription start site (TSS) (10–14). Very limited evidence suggests a possible involvement of genetic variation in such promoters in disease. In 2019, de Lange *et al*. (12) showed that common variants occurring in one *SCN1A* promoter reduce gene expression *in vitro* and influence disease severity in patients with a pathogenic *SCN1A* variant. Haigh *et al*. (15) showed that the deletion of a genomic region containing one *SCN1A* promoter in mice caused a decrease in gene expression and resulted in spontaneous seizures with severe cognitive and behavioural deficits in surviving mice. Although it is known that the regions upstream of the three UEs carry promoter activity, Long *et al*. (11) and Nakayama *et al*. (13) are the only experimental studies that proposed boundaries for the genetic regions carrying *SCN1A* promoter activity in humans. The genetic boundaries proposed in 2010 (13) are still the reference used today by the small number of studies available in the literature, which investigated *SCN1A* promoters experimentally in humans, despite the limitations of that definition that have since emerged: the *SCN1A* promoter associated with the h1c UE was not considered in the study; the length of the genetic regions to test for potential promoter activity was chosen arbitrarily (2.5 kb upstream of h1a and 2 kb upstream of h1b); the experiment was based on an old *SCN1A* gene annotation and genome build (the Human March 2006 NCBI Build 36.1 (hg18) was used) and finally it was not known at the time that TFBSs capable of modulating *SCN1A* expression are also located downstream of the UEs (13). There is currently a lack of a contemporary definition of the boundaries of the genetic regions that either carry promoter activity or might be able to modulate promoter activity and gene expression.

Dong *et al*. (14) also showed that NT2 cells (pluripotent human embryonal carcinoma cells that differentiate into neurons) exclusively use the h1c-associated *SCN1A* promoter, but no study has yet thoroughly clarified the cellular specificity of differential *SCN1A* promoter usage. Further, multiple databases have investigated the genome-wide distribution of gene enhancers, but the genetic location of *SCN1A* enhancers in humans remains unknown (16).

Given the importance of regulatory DNA regions in disease development and modulation, the currently unexplained phenotypic variability of patients with a pathogenic *SCN1A* variant and the lack of a complete understanding of *SCN1A* regulation, here we summarize the current state of knowledge of *SCN1A* regulation, providing details of its promoter and enhancer regions. We then integrate the currently available data on *SCN1A* promoters by extracting information relative to the *SCN1A* locus from genome-wide repositories and attempt to clearly define the genetic regions to be considered when exploring the *SCN1A* regulatory landscape. We explore cellular specificity in the differential usage of *SCN1A* promoters (14). We also review and integrate the available human brain-derived enhancer databases and mouse-derived data in order to provide a comprehensive computationally developed summary of *SCN1A* brain-active enhancers. By querying genome-wide data repositories, extracting *SCN1A*-specific data and integrating the different types of independent evidence, we hope to create a comprehensive catalogue that better defines the regulatory landscape of *SCN1A* and could be used to explore the role of *SCN1A* regulatory regions in disease and treatment response.

## Results

### Definition of *SCN1A* promoter regions

**Figure 1:**
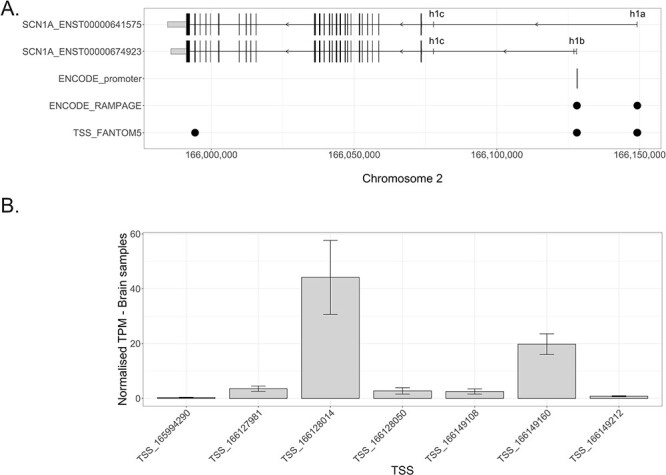
Identification of promoter-like signatures across the *SCN1A* locus. (**A**) The first two tracks indicate the exon–intron structure of the two *SCN1A* transcripts investigated in this paper (i.e. ENST00000641575 and ENST00000674923). The longer vertical lines indicate translated exons, while the shorter vertical lines represent untranslated exons. The width of each line reflects the size of the exon. The direction of transcription of *SCN1A* is indicated by arrows. The three known *SCN1A* promoters, which are associated with three untranslated exons, are indicated as h1a, h1b and h1c. The genetic position of sequence(s) with a promoter-like signature identified by ENCODE (black vertical line; third track) and *SCN1A* TSSs identified by the ENDODE RAMPAGE dataset and FANTOM5 (fourth and fifth tracks, indicated by black circles) are shown. *X*-axis: genetic positions on chromosome 2, *Y*-axis: genetic elements that were considered. (**B**) The relative activity of each *SCN1A* TSS shown as normalized (to the total number of counts) TPM expression. The higher the TPM values represent greater associated TSS activity. The data are derived brain samples present in the FANTOM5 CAGE dataset.

The Encyclopedia of DNA Elements (ENCODE) genome-wide candidate *cis*-regulatory elements (cCREs) v2 registry identified one region with a promoter-like signature (PLS) in close proximity to the h1b UE of *SCN1A*, and TSSs located in the h1a and h1b UEs were identified by the ENCODE RNA annotation and mapping of promoters for the analysis of gene expression (RAMPAGE) dataset (Fig. 1A). The functional annotation of the mammalian genome (FANTOM) 5 cap analysis gene expression (CAGE) dataset identified seven *SCN1A* TSSs: three mapped to the h1a UE, three to the h1b UE and one towards the end of the *SCN1A* transcripts (Fig. 1A). Surprisingly, neither the ENCODE RAMPAGE nor the FANTOM5 CAGE datasets found a TSS mapping in the h1c UE. Given the experimental evidence showing that the genetic region upstream of the h1c UE can drive *SCN1A* expression, the failure to find a TSS in this position could indicate that this region has weaker promoter activity than the promoter regions associated with the h1a and h1b UEs, causing it to be missed by the TSS-identification methods. The measurement of relative TSS activity showed that the TSS located in the coding region of *SCN1A* was not active in the brain; TSS:166128014, in h1b, was the most active, accounting for a mean of 44.1533 tags per million (TPM), indicating that for every 1 000 000 CAGE tags across the genome in the CAGE library, an average of 44.1533 originated from this TSS. The second most active TSS was TSS:166149160, which accounted for a mean of 19.7896 TPM across all brain samples (Fig. 1B).

**Figure 2:**
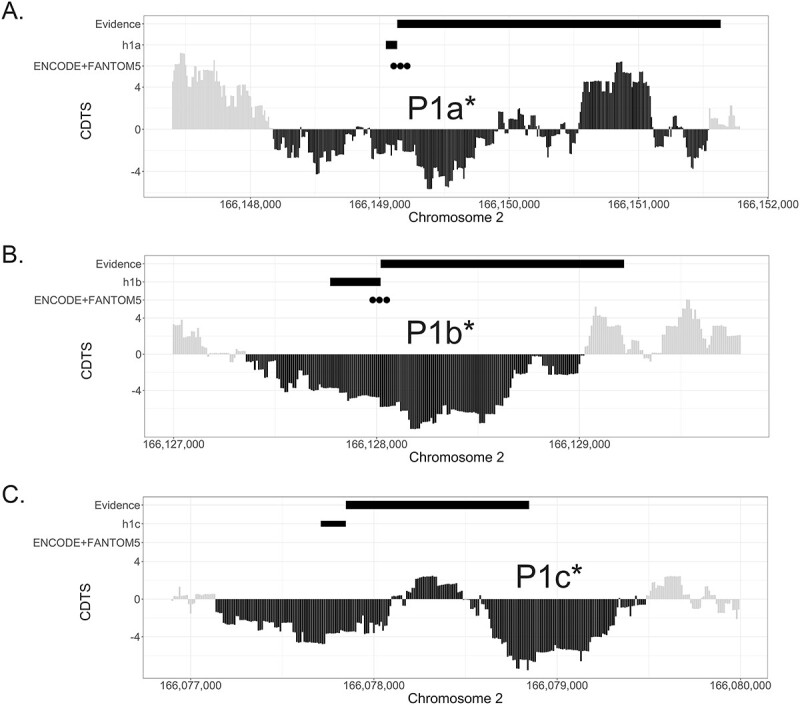
Level of sequence constraint of the genetic region surrounding the untranslated exon h1a (**A**), h1b (**B**) and h1c (**C**). For each panel, the first track highlights the regions for which experimental evidence exists for promoter activity, indicated by the dark grey bar. The second track shows the location of the untranslated exon (h1a, h1b, h1), indicated by a lighter grey bar. ENCODE and FANTOM5 promoter hits are represented as black circles in the third track. Sequence constraint is expressed as CDTS, indicating the likelihood of variation. Negative CDTS scores indicate highly constrained regions, infrequently mutated in healthy individuals and more likely to be functionally relevant. The derived extents of the genetic regions potentially associated with *SCN1A* promoter activity [P1a^*^ (A), P1b^*^ (B), P1c^*^ (C)] are indicated by the horizontal black region of the CDTS, marked by the double headed arrow. The rules for integration of the different data repositories, generating the new promoter signatures, are explained in detail in the results section.

The map of sequence constraint across the human genome created by di Iulio *et al*. (33) showed that the three UEs, the sequences with a promoter-like epigenetic signature identified by ENCODE and the *SCN1A* TSS identified by the ENCODE RAMPAGE dataset and FANTOM5, fall within broader regions of the human genome that are highly constrained, indicating that they are rarely mutated in healthy individuals and therefore likely to be functional (Fig. 2A–C). We then integrated the available experimental data around promoter activity in the *SCN1A* 5′ untranslated region (UTR) region. Long *et al*. (11) demonstrated the promoter activity of the 2.5 kb region upstream of h1a, findings confirmed by Nakayama *et al*. (13), who also identified strong promoter activity from the 1.2 kb region upstream of h1b. Dong *et al*. (14) demonstrated the promoter activity of a construct containing the 1 kb region upstream of h1c (13,14) (Fig. 2A–C). By combining these data, we defined the regions associated with *SCN1A* promoter activity as those which included the hits from the two promoter databases used, the regions with experimentally proven promoter activity and those with a high level of constraint. In defining these regions, priority was given to negative context-dependent tolerance score (CDTS) scores; regions with positive CDTS were included if they were encompassed by negative CDTS and overlapped with experimental evidence of promoter activity. Furthermore, as experimental evidence has shown that the three UEs contain multiple TFBSs capable of modulating *SCN1A* expression, and haplotypes located within h1a and in the upstream promoter region also influence *SCN1A* expression levels *in vitro*; we marked the regions downstream of the three UEs as potentially relevant for *SCN1A* promoter functionality, thus including the core and proximal promoter regions, but also potential downstream TFBSs (11,12,14). We defined the regions associated with *SCN1A* promoter activity as P1a^*^ (GRCh38.p13:166148180-166151550), P1b^*^ (GRCh38.p13:166127360–166129030) and P1c^*^ (GRCh38.p13:166077140–166079490) (Fig. 2A–C).

**Figure 3:**
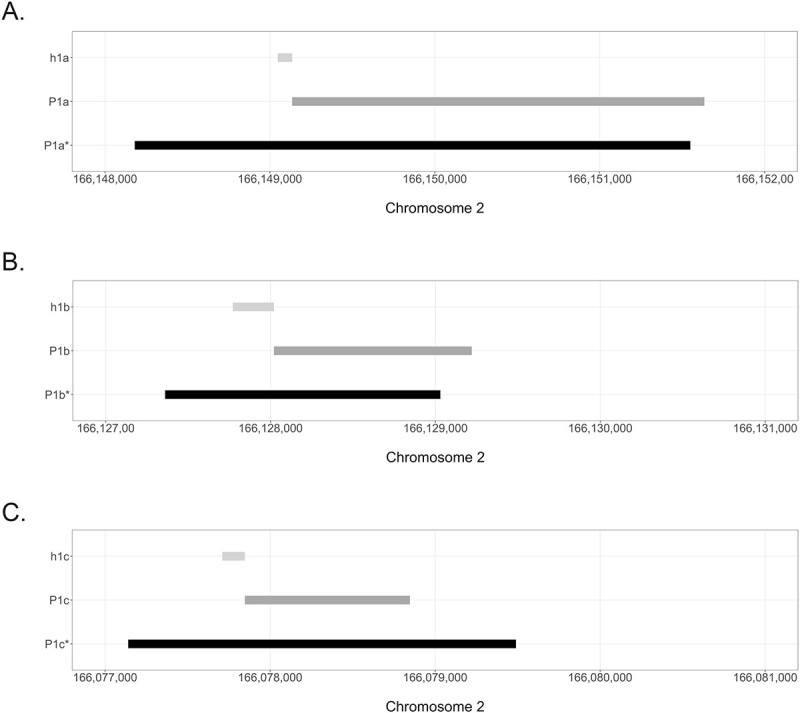
Changes from the previously defined promoter regions, P1a, P1b and P1c, to the novel definition of genetic regions associated with *SCN1A* promoter activity. Each panel shows the location of the untranslated exons h1a, h1b and h1c (indicated in light grey, top track in each panel), the previous definition of P1a, P1b and P1c (indicated in dark grey, middle track in each panel) and the novel definition of promoter activity of P1a^*^, P1b^*^ and P1c^*^ (indicated in black, bottom track in each panel), all showing the changes resulting from the analysis undertaken in this work. (**A**) P1a^*^ (GRCh38.p13:166148180–166 151 550). (**B**) P1b^*^ (GRCh38.p13:166127360–166129030). (**C**) P1c^*^ (GRCh38.p13:166077140–166079490).

A few studies that investigated *SCN1A* promoters in humans used the genetic boundaries proposed by Nakayama *et al*. (13) as references for the h1a and h1b UEs associated promoters, commonly referred to as P1a and P1b promoters. However, there are numerous limitations with this definition: the *SCN1A* promoter associated with the h1c UE was not considered; the length of the genetic regions to test for potential promoter activity was chosen arbitrarily; the experiment was based on an old *SCN1A* gene annotation and genome build (Human March 2006 NCBI Build 36.1: hg18) and most importantly the presence of TFBSs capable of modulating *SCN1A* expression located downstream of the UEs were not known (12,14). Our novel proposition of boundaries for genetic regions associated with *SCN1A* promoter activity (P1a^*^, P1b^*^ and P1c^*^), generated by integrating the most up to date knowledge on genetic regions potentially relevant for *SCN1A* promoter functionality (including core and proximal promoter regions, but also potential downstream TFBSs), as well as computationally derived information, results in a change in the previously defined P1a and P1b, as proposed by Nakayama *et al*. and P1c, as proposed by Dong *et al*. (13,14) (Fig. 3).

### Definition of *SCN1A* enhancer regions

**Figure 4:**
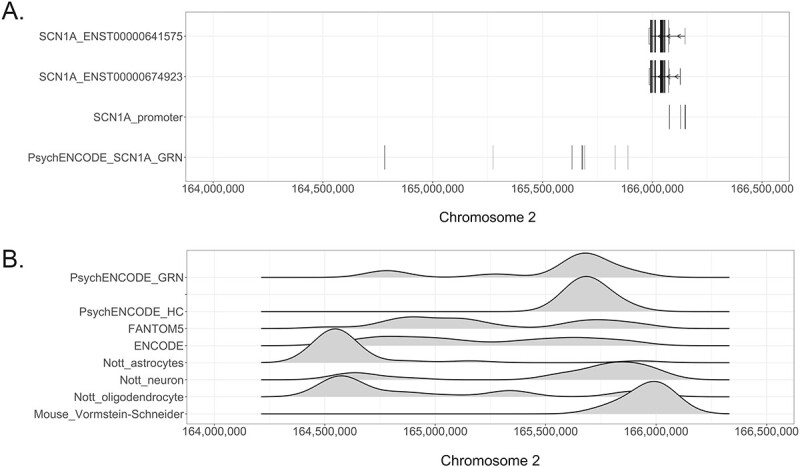
Identification of *SCN1A* enhancer regions. (**A**) Genetic position of two *SCN1A* protein-coding transcripts (ENST00000641575 and ENST00000674923). The longer vertical lines indicate translated exons, while the shorter vertical lines represent untranslated exons (first and second tracks). The width of each line reflects the size of the exon. The direction of transcription of *SCN1A* is indicated by arrows. The third track, ‘SCN1A_promoter’, shows the position of the new regions putatively associated with *SCN1A* promoter activity (P1a^*^, P1b^*^, P1c^*^). The last track shows *SCN1A* enhancer regions, as defined by the PsychENCODE GRN dataset (all indicated by vertical lines). *X*-axis: genetic positions on chromosome 2, *Y*-axis: genetic elements that were considered. (**B**) Genetic sequences with enhancer-like signature identified in the *SCN1A* locus by multiple independent databases, including the PsychENCODE GRN dataset, PsychENCODE high-confidence (HC) enhancer dataset, the FANTOM5 and ENCODE enhancer repositories, the cell-type-specific enhancer datasets produced by Nott *et al*. (18) and the mouse-derived *Scn1a* enhancer dataset produced by Vormstein-Schneider *et al*. (17). The height of the profile is indicative of the likelihood that a region is associated with enhancer activity based on the interpretation of the named datasets. The *y*-axis is unitless, and comparison should only be made within the same track, rather than across multiple tracks.

The genetic position of the *SCN1A* enhancer regions was identified by integrating multiple databases and data repositories. By querying the PsychENCODE gene regulatory network (GRN) for genetic sequences marked as brain active *SCN1A* enhancers, nine enhancer regions were identified (Fig. 4A). Seven enhancers were located at around 250 kb from the *SCN1A* coding sequence (CDS), and two were located over 700 kb from the *SCN1A* gene. The genetic region containing the seven enhancers that were closer to *SCN1A* was also identified as an enhancer-like region by all genome-wide datasets interrogated and also supported by the mouse-derived dataset (Fig. 4B), whereas the region containing the two enhancers at over 700 kb by the *SCN1A* gene was not recognized as an enhancer-like region from the PsychENCODE high-confidence dataset and the mouse-derived dataset but was marked as an enhancer region in the other datasets queried (Fig. 4B).

**Figure 5:**
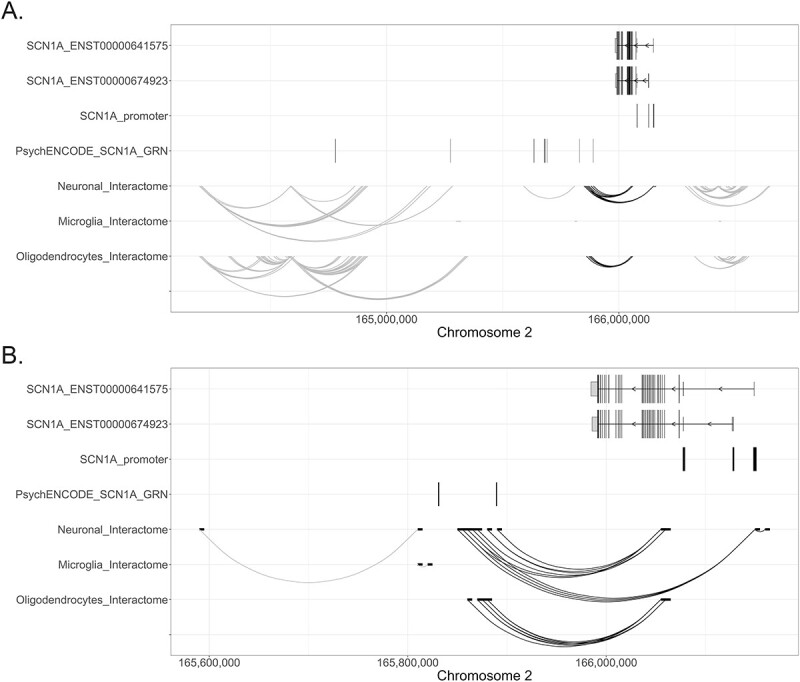
Gene promoter–enhancer interactions in the *SCN1A* gene locus. (**A**) Genetic position of two *SCN1A* protein-coding transcripts (ENST00000641575 and ENST00000674923) (top two tracks). The longer vertical lines indicate translated exons, while the shorter vertical lines represent untranslated exons. The width of each line reflects the size of the exon. The direction of transcription of *SCN1A* is indicated by arrows. The location of the *SCN1A* promoters (P1a^*^, P1b^*^, P1c^*^) and *SCN1A* enhancer regions, as defined by the PsychENCODE GRN dataset are indicated by vertical lines in tracks 3 and 4, respectively. Chromatin interactions were derived from the cell-type-specific genome-wide interactome datasets produced by Nott *et al*. (18), which includes gene promoter–enhancer interactions experimentally identified in neurons, oligodendrocytes and microglia (17). Chromatin interactions in the broader *SCN1A* locus across different brain-relevant cell-types are shown in (A), and a zoomed-in view of the interactions occurring between the putative *SCN1A* enhancers and *SCN1A* promoters is shown in (**B**). The region of interaction is indicated by the bold black segment at the terminal end of each curve.

Next, we assessed whether the regions identified as *SCN1A* enhancers by the PsychENCODE GRN dataset were linked to *SCN1A* in other independent databases. We used the cell-type-specific genome-wide interactome datasets produced by Nott *et al*. (17) extracted the data relative to the *SCN1A* locus and explored whether the potential enhancer regions physically interact with the *SCN1A* promoters. The analysis showed that the putative *SCN1A* enhancers located farthest away from the *SCN1A* gene body did not interact with the *SCN1A* promoters, whereas the genetic region containing the two putative enhancers that are closest to the gene showed multiple interactions with the *SCN1A* promoter regions (Fig. 5A). Multiple gene promoter–enhancer interactions were discovered in the neuronal dataset between the genetic region containing the two putative enhancers closest to the gene and the P1a^*^ and P1c^*^  *SCN1A* promoters; chromatin interactions were found in the oligodendrocyte dataset between the genetic region containing the two putative enhancers closest to the gene and the P1c^*^ promoter and no gene promoter–enhancer interactions were observed in the microglia dataset (Fig. 5B). The integration of data from multiple enhancer datasets and the cell-type-specific promoter–enhancer interactome datasets supported the presence of *SCN1A* enhancers located around 125 kb from the CDS of the gene, interacting with two of the three *SCN1A* promoters.

Although the genetic location of the various *SCN1A* regulatory elements is addressed here, the final *SCN1A* transcript levels are determined by the combined activity of such regulatory regions, and there is currently no computational method capable of predicting how the activity of each regulatory element is weighted, integrated and how they function collectively.

### Cell specificity

**Figure 6:**
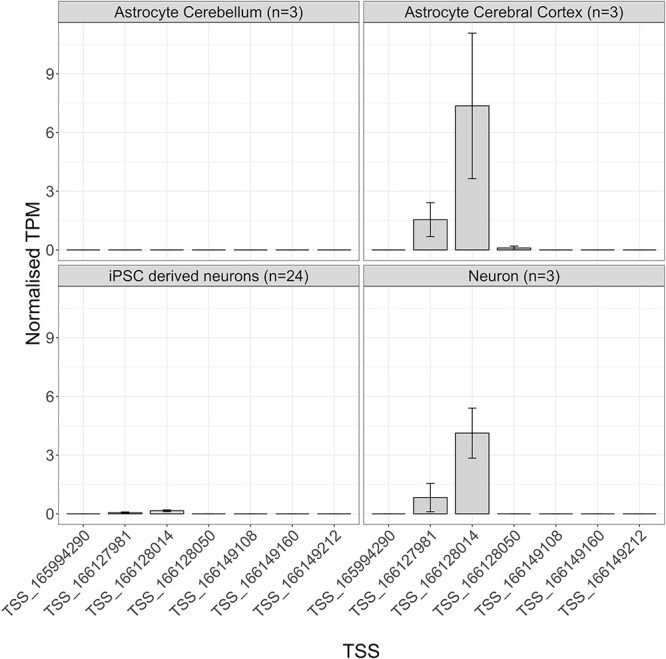
Normalized TPM expression values of *SCN1A* TSSs across different cell-types. Clockwise from the top-left panel: primary astrocytes from the cerebellum (*n* = 3); primary astrocytes from the cerebral cortex (*n* = 3), primary neurons (*n* = 3) and iPSC-derived neurons (*n* = 24). The black vertical bars indicate the standard error (SE) of the mean. Higher TPM values reflect greater associated TSS activity.

To explore the differential usage of *SCN1A* promoters across brain cell types, we first compared the relative activity of *SCN1A* TSSs across brain cells using the CAGE dataset produced by FANTOM5. There was no activity at any of the seven SCN1A TSSs in astrocytes isolated from cerebellum tissue or induced pluripotent stem cells (iPSCs) differentiated into neurons; in astrocytes isolated from the cerebral cortex and neurons, TSS:166128014, in P1b^*^, was the most active, accounting for a mean of 7.3615 TPM in astrocytes and 4.1252 TPM in neurons, and the second most active TSS was TSS:166127981, in P1b^*^, which accounted for a mean of 1.5472 TPM in astrocytes and 0.8320 TPM in neurons (Fig. 6). Only astrocytes isolated from the cerebral cortex showed activity from TSSs located in P1a^*^, with a mean of 0.0994 TPM detected from TSS:166128050 (Fig. 6).

**Figure 7:**
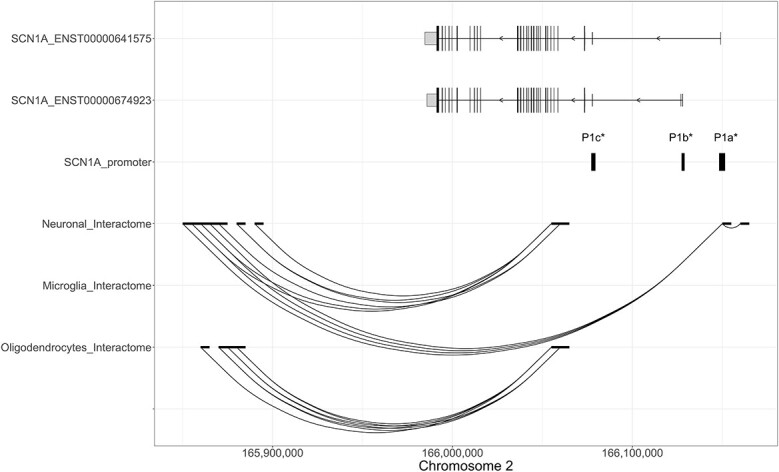
Gene promoter–enhancer cell-type-specific interactions involving the three *SCN1A* promoter regions. The first two tracks indicate the exon–intron structure of the two *SCN1A* transcripts investigated in this study (i.e. ENST00000641575 and ENST00000674923) (top two tracks). The longer vertical lines indicate translated exons, while the shorter vertical lines represent untranslated exons. The width of each line reflects the size of the exon. The direction of transcription of *SCN1A* is indicated by arrows. The third track indicates the location of the sites with putative promoter activity (P1a^*^, P1c^*^, P1b^*^). Finally, in the bottom three tracks, the cell-type-specific (neurons, microglia and astrocytes) gene promoter–enhancer interactions relative to P1a^*^, P1c^*^, P1b^*^ are shown. The strength of each interaction is not shown.

To further explore the presence of cellular specificity in the usage of *SCN1A* promoters, we used the Nott *et al*. (17) cell-type-specific interactome datasets and compared promoter–enhancer interactions occurring in different cell types. The neural interactome revealed gene promoter–enhancer interactions between enhancers and the P1a^*^ promoter, as well as close to the P1c^*^ promoter, whereas the oligodendrocyte interactome revealed only promoter–enhancer interactions with a genetic region near the P1c^*^ promoter (Fig. 7).

## Discussion

Gene promoters and enhancers are critical in the modulation of gene expression, and genetic variation in such regulatory elements has an established role in human disease, with growing evidence from multiple fields (6,7,18). Several studies have investigated and predicted the genome-wide distribution of non-coding regulatory regions, but only a few have provided experimental evidence of the regulatory landscape and only for a limited number of genes of interest. In the case of *SCN1A*, despite being probably the single most important gene in epilepsy, both responsible for multiple epileptic syndromes and the target of several experimental gene-based therapies, little is known about its regulatory landscape (1). Phenotypic heterogeneity is a typical feature of *SCN1A*-related epilepsies and remains largely unexplained: genetic variation in the non-coding regulatory elements that modulate *SCN1A* expression could be one potential causal factor (19,20). However, the lack of a clear definition of the *SCN1A* regulatory landscape precludes such investigation. Here, combining the existing evidence on *SCN1A* promoters from Long *et al*. (11), Nakayama *et al*. (13) and Dong *et al*. (14) and integrating data relative to *SCN1A* extracted from contemporary genome-wide promoter and enhancer data repositories, we proposed boundaries of the regions putatively associated with promoter activity or modulation of promoter activity. Using the Nott *et al*. (17) genome-wide cell-type-specific interactome dataset and extracting information on the *SCN1A* locus, we found that both the P1a^*^ and P1c^*^ promoters seem to be active in neurons, whereas in oligodendrocytes, the P1c^*^ promoter appears to be the main promoter driving *SCN1A* expression. In astrocytes, according to the FANTOM5 database, the P1b^*^ is the only active promoter. By integrating multiple independent databases, we also showed the presence of *SCN1A* enhancers located around 125 kb downstream of the CDS of the gene, which however requires experimental validation.

Our computational definition of the *SCN1A* regulatory elements could be used as a foundation when exploring the status of the individual *SCN1A* regulatory landscape variation in patients with a pathogenic variant in *SCN1A* or for other epilepsies for which the use of sodium channel blockers has been proven useful, such as *KCNQ2*-related encephalopathies (21). The information on the differential usage of *SCN1A* promoters across brain cell types could be useful in the development of treatment strategies that target different promoter regions in different cell types to rescue or modulate *SCN1A* expression. Further, the high level of TSS activity observed in astrocytes isolated from the cerebral cortex and neurons supports the research focus on neurons as being the crucial cell type involved in the pathology of *SCN1A*-related epilepsy but also highlights the currently under-explored potential role of cortex-derived astrocytes. Astrocytes are involved in the sensing and modulation of neuronal activity through the uptake and release of neurotransmitters, and recent studies have shown that abnormal astrocyte function can influence the development and aggravation of epilepsy (22,23). Astrocytes may thus also become justifiable targets for novel therapeutic strategies for *SCN1A*-related and other epilepsies.

However, the analysis of the *SCN1A* regulatory landscape and its potential involvement in the clinical presentation of patients is hampered by difficulties in validation. In fact, when multiple genetic variations in gene promoters and enhancers are discovered, it is extremely difficult to determine what the combined outcome of those variants might be in terms of gene expression. This is further compounded by the complexity of the path from gene to phenotypes and the multiple potential regulatory mechanisms that occur at the transcriptional, post-transcriptional, translational and post-translational level. For example, in this study, we did not investigate potential miRNA or long non-coding regulation of *SCN1A*. It is likely that these elements do impact on the expression of *SCN1A* and the final associated phenotype; however, the molecular mechanisms underlying these regulations remain poorly understood so were not included in the paper. In the future, these regulatory elements should be investigated in more depth. Ultimately, the creation of a computational tool that can forecast the effects of regulatory elements, including miRNA and long non-coding RNAs, on gene expression would be one aim of this work. Nevertheless, the derived regions presented here will hopefully provide a starting point for such endeavours.

## Materials and Methods

### *SCN1A* gene transcripts

**Figure 8:**
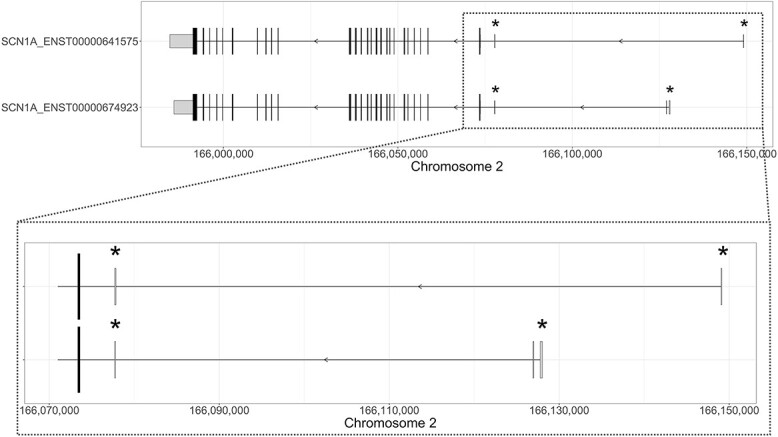
*SCN1A* protein-coding transcripts used as references. The exon–intron structure of the two *SCN1A* transcripts investigated in this study (i.e. ENST00000641575 and ENST00000674923) is shown. The longer vertical lines indicate translated exons, while the shorter vertical lines represent UEs. The width of each line reflects the size of the exon. The direction of transcription of *SCN1A* is indicated by arrows. Asterisks indicate UEs associated with promoter activity. The bottom inset zooms in on the untranslated exons of both *SCN1A* transcripts.

Among the protein-coding transcripts, we selected the longest transcripts that showed the different *SCN1A* UEs associated with promoter activity and were the most well supported across different databases. ENST00000674923.1 is the best supported *SCN1A* protein-coding transcript, contains the h1b and h1c UEs and is the reference transcript in the Matched Annotation from the NCBI and EMBL-EBI (MANE) Select database, which identifies gene transcripts that are identical between the RefSeq and Ensembl/GENCODE databases for UTRs, CDS and splicing and are highly expressed and conserved (24). ENST00000674923.1 is also the *SCN1A* Ensembl Canonical transcript, indicating the most conserved and most highly expressed transcript with the longest CDS that is identical to other resources, such as NCBI and UniProt databases; it is a member of the GENCODE basic gene set, which prioritizes full-length protein-coding transcripts and is classified as principal transcript 4 by APPRIS (25–27). APPRIS is a database that annotates alternatively spliced transcripts to identify the most functionally important ones and identifies the main and alternative isoforms for each gene (27). ENST00000641575.1 is the longest *SCN1A* protein-coding transcript; it contains the h1a and h1c UEs, belongs to the GENCODE basic gene set, and is classified by APPRIS as alternative transcript 1, which denotes a gene transcript that is conserved across at least three species (27). The GRCh38:ENST00000674923 and GRCh38:ENST00000641575 *SCN1A* transcripts were used as references (Fig. 8).

### Definition of *SCN1A* promoter regions

The *SCN1A* promoter regions associated with the three UEs h1a (or h1u), h1b and h1c (or h2u) were studied. To define the boundaries of the regulatory regions associated with promoter activity or modulation of promoter activity, we collected data from the ENCODE project, including the cCREs v2 registry and the RAMPAGE TSS datasets, the FANTOM 5 data repository, human sequence constraint data, extracted the *SCN1A*-related records and integrated the resulting information with experimental data pulled from the available literature.

From the candidate cCREs v2 registry, generated by the ENCODE project, the genomic coordinates of sequences with a PLS, which were defined as genetic regions with high DNase signals and high H3K4me3 signals, were selected (28). From the ENCODE repository, the RAMPAGE TSS collection was also interrogated (29). RAMPAGE is a sequencing method that captures the 5′ end of capped RNAs using paired-end reads to identify TSSs throughout the genome at base-pair resolution. From the ENCODE RAMPAGE database, TSSs located in the *SCN1A* locus identified in human brain tissue samples, including the temporal lobe (isolated from two embryos of 20 and 24 post conception weeks (PCW)), parietal lobe (isolated from two embryos of 22 and 24 PCW), occipital lobe (isolated from two embryos of 20 and 22 PCW), frontal cortex (isolated from two embryos of 20 and 22 PCW) and cerebellum (isolated from two embryos of 19 and 37 PCW), were retrieved (29). The genetic location of TSSs associated with *SCN1A* expression was also obtained from the FANTOM5 data repository. The FANTOM5 project performed CAGE across 975 samples, including human primary cells, tissue samples and cancer cell lines and mapped TSSs throughout the genome (30–32). From the same data repository, the relative activity of each TSS, measured as normalized (to the total number of counts) TPM and calculated using the relative log expression method in edgeR, was collected for all brain samples examined in the FANTOM5 CAGE dataset (30–32). The map of sequence constraint for the human genome created by di Iulio *et al*. (33) was used to identify sequences that are rarely mutated in healthy individuals, intolerant to variation and thus more likely to be functionally relevant. The map, which was produced using whole-genome sequencing data from 11 257 individuals, assigns a CDTS to each 10 bp long bin of the genome, indicating the likelihood of variation: the lower the score, the less frequently the bin is affected by variation and the more mutation intolerant the bin is (33). As it is known assumptions concerning conservation differ between protein-coding and non-coding regions of the genome, and as it has been previously shown that CDTS can be used to detect non-coding regulatory regions in humans (33), the CDTS rather than the conservation score was used to assess intolerance to mutation over the regions of interest. The available literature on *SCN1A* promoter regulation was also reviewed.

### Definition of *SCN1A* enhancer regions

To identify the genetic position of the *SCN1A* enhancer regions, multiple databases and data repositories were compared and integrated. As a starting point for recognizing potential *SCN1A* enhancers, the PsychENCODE GRN dataset was queried, and the genetic regions marked as brain-active *SCN1A* enhancers were selected. The PsychENCODE GRN dataset was produced by integrating transcription factor binding site analysis, the full PsychENCODE enhancer dataset, quantitative trait loci (QTLs) and Hi-C data. However, considering that in the case of *SCN1A*, a very limited number of QTLs were identified (4 eQTLs, 6 isoQTLs, 0 tQTLs) and the fact that Hi-C identifies chromatin interactions at kilobase resolution, we exploited additional datasets to confirm that the regions marked as *SCN1A* enhancers by the PsychENCODE GRN dataset were marked as enhancer-like regions by other studies and, if so, they were linked to *SCN1A*.

To increase the confidence that the genetic regions marked as *SCN1A* enhancers by the PsychENCODE GRN dataset were marked as enhancer-like regions by other studies, we explored multiple genome-wide datasets, including the PsychENCODE high-confidence enhancer dataset, the FANTOM5 enhancer dataset, the sequences with a distal enhancer-like signature from the cCREs ENCODE registry v2, the cell-type-specific enhancer datasets produced by Nott *et al*. (17) and the mouse-derived *SCN1A* enhancer dataset produced by Vormstein-Schneider *et al*. (16,17,28,34–36). Then, to test whether the regions identified as *SCN1A* enhancers by the PsychENCODE GRN dataset were actually linked to *SCN1A* in other independent datasets, we queried the cell-type-specific genome-wide interactome datasets produced by Nott *et al*. (17), which includes gene promoter–enhancer interactions experimentally identified in neurons, oligodendrocytes and microglia and extracted the data relative to *SCN1A*. The Nott *et al*. (17) interactome datasets were produced using proximity ligation-assisted ChIP-seq, which performs a high-resolution mapping of long-range chromatin interactions (37).

### Cell specificity

To investigate the differential usage of *SCN1A* promoters across brain cell types, we first extracted the *SCN1A*-related data obtained from primary brain cells examined in the FANTOM5 CAGE dataset (30–32). The selection resulted in 33 samples being considered: three neuronal cells, three astrocytes extracted from cerebellar tissue, three astrocytes extracted from cerebral cortex tissue and 24 iPSCs at different stages of differentiation into neurons. Then, to explore further the usage of *SCN1A* promoters across different cell types, we used the Nott *et al*. (17) genome-wide cell-type-specific interactome datasets, extracted the information relative to *SCN1A* and compared the gene promoter–enhancer interactions between different cell types.

## Data Availability

Data sharing is not applicable to this article as no new data were created for this study. All datasets used this manuscript were derived form publicly available resources. For full details please see the methods.
