# Correction to: SCN1A: bioinformatically informed revised boundaries for promoter and enhancer regions

**DOI:** 10.1093/hmg/ddad071

**Published:** 2023-05-02

This is a correction to: Susanna Pagni, Helena Martins Custodio, Adam Frankish, Jonathan M Mudge, James D Mills, Sanjay M Sisodiya, SCN1A: bioinformatically informed revised boundaries for promoter and enhancer regions, *Human Molecular Genetics*, 2023;, ddad015, https://doi.org/10.1093/hmg/ddad015

In the originally published version of this manuscript, Figure 5 and Figure 7 erroneously omitted the chromatin interactions observed in neurons.

This error has been corrected.

